# 2509: Estimation of HIV viral load using quantitative measurement of HIV-p24 on lateral flow immunoassays

**DOI:** 10.1017/cts.2017.53

**Published:** 2018-05-10

**Authors:** Joseph A. Conrad, Kelly Richardson, Anna Bitting, Spyros Kalams, David Wright

**Affiliations:** Vanderbilt University, Nashville, TN, USA

## Abstract

OBJECTIVES/SPECIFIC AIMS: High-sensitivity diagnostics for early infant diagnosis (EID) of HIV at the point of care (POC) are not widely available. Lateral flow immunoassays (LFA) can detect HIV-p24, but are not sensitive enough in practice. With improvements, LFA are a compelling platform for POC in EID. We used functionalized magnetic beads and immunocomplex dissociation to improve sensitivity of HIV-p24 LFA. Here, we evaluate the utility for LFA to quantitatively report HIV-p24 concentration and estimate HIV viral load. Using purified p24 protein and virion constructs, we determined the limits of detection for HIV-p24 using LFA rapid tests. Using measurements from HIV-p24 ELISA, laboratory-developed RT-qPCR, droplet digital PCR, and gold standard clinical viral load, we further characterized the relationship between HIV-p24 concentration, HIV genomic RNA, and LFA test line signal. METHODS/STUDY POPULATION: We measured HIV-p24 concentration by ELISA (R&D Systems) and LFA (Alere Determine HIV-1/2 Ab/Ag Combo). An LFA reader instrument was used to image test lines and measure test line signal on the LFA. HIV viral loads were measured using RT-qPCR and droplet digital RT-PCR protocols adapted in our lab. We obtained gold standard viral load measurements using the Roche Cobas TaqMan system at Vanderbilt University Medical Center. Data analysis was performed using Prism 7 and Stata 14. RESULTS/ANTICIPATED RESULTS: LFA test line signal increases in a predictable, dose-dependent manner and correlates with concentration of purified HIV-p24 with a linear range between 50 and 1000 pg/mL (Spearman *r*=1; *p*=0.0004). We compared p24 concentration (ELISA). We evaluated the utility of LFA to quantify HIV-p24 from virions suspended in human plasma, which increased the limit of detection for HIV-p24 to 100 pg/mL and shifted the linear range 100–10,000 pg/mL (Spearman *r*=0.77; *p*<0.001). To evaluate the relationship between HIV-p24 concentration and concentration of HIV RNA, we employed 3 molecular techniques. The LFA is capable of detecting HIV-p24 concentrations that correspond to a range of viral loads between 653,000 and 1655 copies of viral RNA/mL. DISCUSSION/SIGNIFICANCE OF IMPACT: Our preliminary results are very promising, indicating that commercially available LFA can quantitatively measure HIV-p24 concentration to low levels. When coupled with our analysis of the relationship between HIV-p24 concentration and HIV RNA concentration, LFA may be a potential platform allowing us to estimate HIV viral burden at clinically relevant levels. Our next steps will be to evaluate this relationship in primary, clinical specimens in collaboration with the Tennessee Center for AIDS Research. We will incorporate technologies to improve the sensitivity of these LFA and evaluate their performance in field settings in Zambia. Our findings are broadly applicable for use in HIV care and treatment programs and early infant diagnosis programs around the world.

